# A Unique Variation of Quadratus Plantae in Relation to the Tendons of the Midfoot

**DOI:** 10.3390/jfmk7020049

**Published:** 2022-06-09

**Authors:** Lokesh A. Coomar, Daniel T. Daly, Jay Bauman

**Affiliations:** Center for Anatomical Science and Education, Department of Surgery, Saint Louis University School of Medicine, Saint Louis, MO 63104, USA; lokesh.coomar@slu.edu (L.A.C.); daniel.daly@health.slu.edu (D.T.D.)

**Keywords:** quadratus plantae, flexor digitorum accessorius, flexor hallucis longus, flexor digitorum longus, chiasma plantare, master knot of Henry, foot, anatomical variation

## Abstract

A novel combination of variations involving the quadratus plantae muscle (QP) and its relationship to the flexor hallucis longus (FHL) tendon was observed unilaterally in the right foot of an 88-year-old female cadaver during routine dissection. The medial head of QP was observed inserting onto the tendon of FHL rather than the tendon of flexor digitorum longus (FDL), while also contributing to an anomalous tendinous slip to the second digit in conjunction with the tendon of FHL. The tendon of FHL also gave off a slip to the third digit. Both tendinous slips attached distally to the digital tendons of FDL. Lastly, the lateral head of QP inserted onto the tendinous slip from FHL to the third digit. Ninety-five additional feet were assessed for these variations, but none were observed. This combination of variations expands upon the proposed actions of QP in the literature. Furthermore, connections between the tendons of the midfoot are of clinical significance for harvesting tendon grafts.

## 1. Introduction

Quadratus plantae, also known as the flexor digitorum accessorius, is a flat, quadrilateral muscle found in the second layer of the intrinsic muscles of the plantar foot. It has no analogous muscle in the hand [1,2,3]. Typically, this muscle has two heads: the medial head, which originates on the medial plantar surface of the calcaneus, and the lateral head, which arises from the lateral border of the calcaneus [4,5]. Both heads of QP converge to insert onto the posterolateral border of the tendon of FDL [4,5]. Several potential actions of QP have been proposed, including countering the oblique pull of FDL, stabilizing the lumbricals, and acting as a lesser flexor of the second through fifth digits [1,4,6].

Previous reports regarding the insertion of QP show variability, but traditionally it inserts strictly on FDL [3,6]. Moreover, the tendon of FHL is reported to give off accessory tendinous slips that insert variably on the second, third, and fourth digits [5,6]. However, in all reported cases involving accessory tendinous slips from FHL, QP inserts on FDL or both FDL and FHL, but not exclusively on FHL [1,6,7,8].

Interconnections between the tendons of FDL and FHL were first described by Arnold Kirkpatrick Henry in 1945 as the master knot of Henry, and have since become known as the chiasma plantare [7]. The chiasma plantare is described as an intersection between the tendons of FDL and FHL where they cross in the midfoot [9]. This relationship serves as an important landmark in tendon harvesting for grafting procedures [7,10]. Since Henry’s initial description, multiple classification systems have been proposed to describe variations in the connections between both tendons [7,8,11].

This report describes a novel QP insertion onto both the FHL tendon and variant tendinous slips to the second and third digits. This unique combination of variations presents an opportunity to re-evaluate the proposed actions of QP given its independence from FDL and the lumbricals.

## 2. Case Presentation

A novel variation involving QP and FHL was noted in the right foot of an 88-year-old female cadaver during routine dissection. Ninety-five additional feet were examined for similar variations, but none were observed. All bodies were received through the Saint Louis University (SLU) Gift of Body Program of the Center for Anatomical Science and Education (CASE) with signed informed consent from the donors. The CASE gift body program abides by all rules set forth by the Uniform Anatomical Gift Act (UAGA).

**Figure 1:**
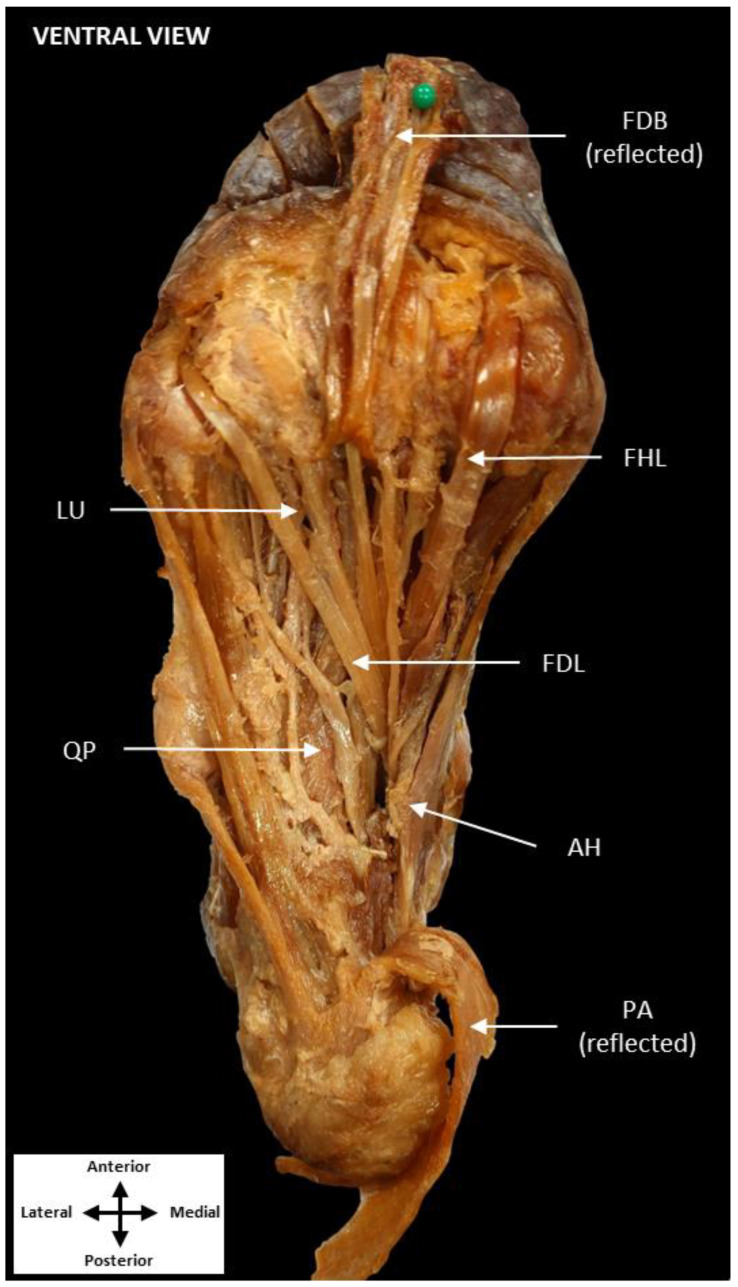
Dissection of the plantar surface of the foot. Dissection of the medial and central compartments of the plantar foot showing the QP diving deep to the FDL. (Plantar aponeurosis: PA, flexor digitorum brevis: FDB, abductor hallucis: AH, flexor digitorum longus: FDL, flexor hallucis longus: FHL, lumbrical muscles: LU, quadratus plantae: QP).

**Figure 2:**
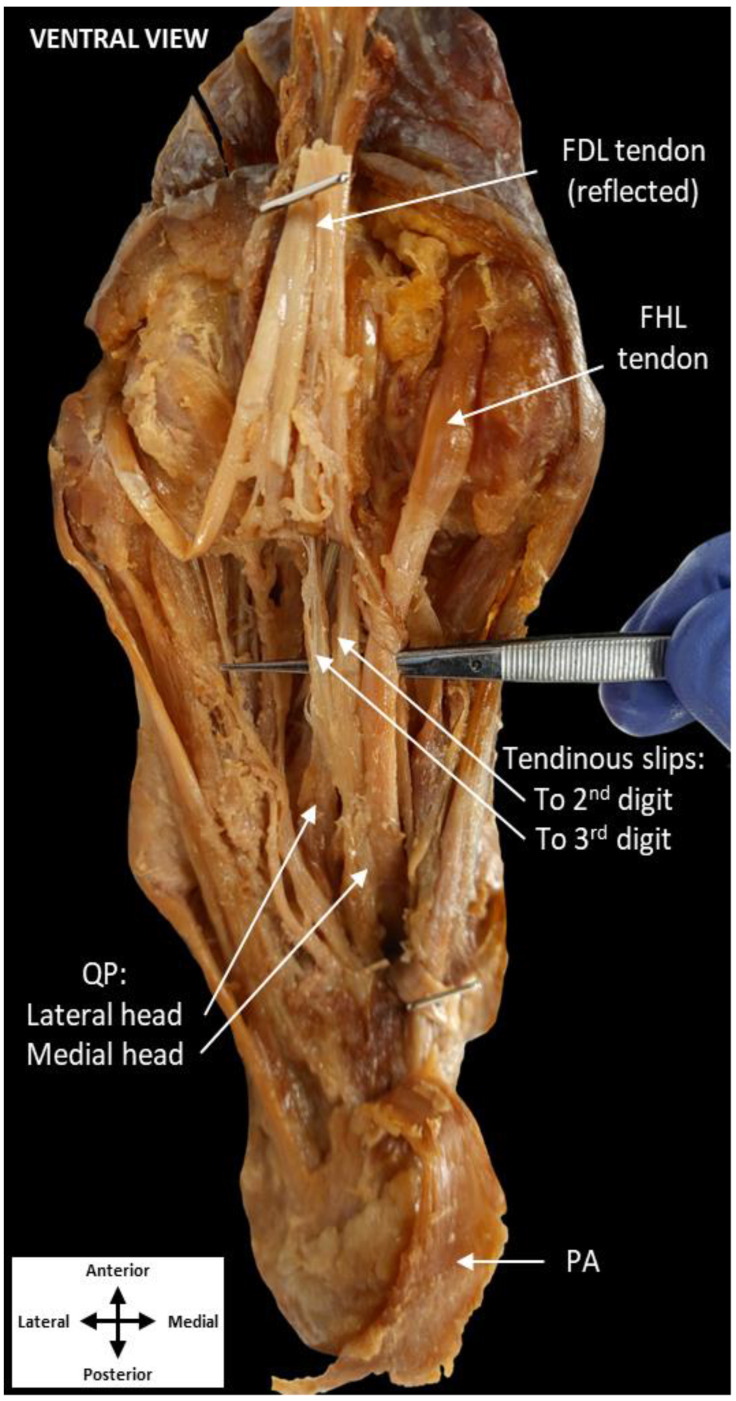
Reflection of the tendon of FDL and tendinous slips. Two tendinous slips were observed inserting along the second (1st slip) and third (2nd slip) digits. These slips are separate from the tendon of FDL (reflected), FHL, and the lumbricals. Additionally, a portion of the medial head of QP inserts on the tendon of FHL, while the lateral head of QP inserts on the tendinous slip to the third digit. (plantar aponeurosis: PA, flexor digitorum longus: FDL, quadratus plantae: QP, flexor hallucis longus: FHL).

All dissection procedures were performed per Grant’s Dissector [12]. The QP muscle was revealed following removal of the skin, superficial fascia, plantar aponeurosis, abductor hallucis, and flexor digitorum brevis (Figure 1). Quadratus plantae was found traveling deep to the FDL tendon rather than inserting on it, as is typically observed. To follow QP toward its insertion, the FDL tendon and lumbricals were reflected, revealing both the lateral and medial heads of QP in close approximation to the FHL tendon (Figure 2). Additionally, two tendinous slips appeared to originate from the medial head of QP. These slips inserted distally on the tendons of FDL to the second and third digits just proximal to the metatarsophalangeal joints.

**Figure 3:**
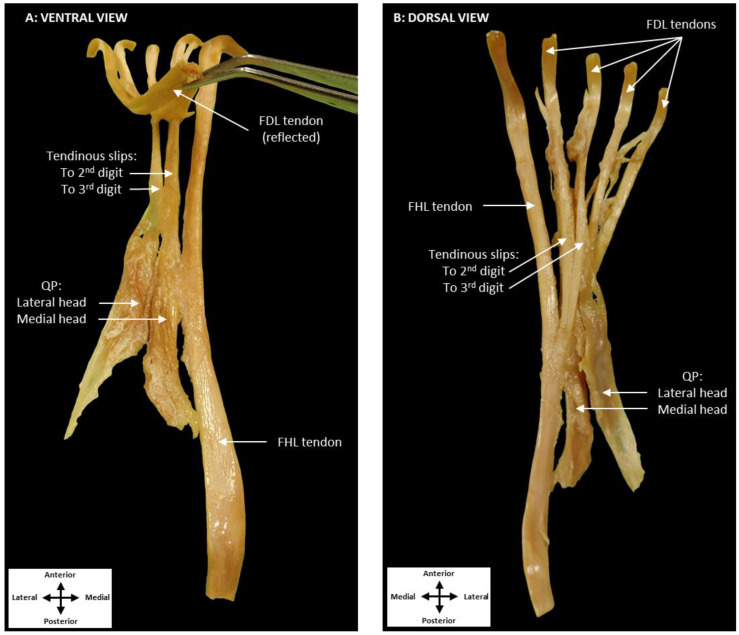
Dissection of QP, the tendon of FDL, and the tendon of FHL. The ventral view (**A**) shows the medial head of QP contributing to the first tendinous slip. The lateral head of QP inserts onto the second tendinous slip. Additionally, the tendinous slips to the second and third digits are attaching to the tendons of FDL. The dorsal perspective (**B**) shows the FHL tendon contributing to both the first and second tendinous slips. (Flexor digitorum longus: FDL, quadratus plantae: QP, flexor hallucis longus: FHL).

To further explore this variation, the QP muscle and tendons of the FHL and FDL were isolated (Figure 3). Ventrally, the medial head of QP was observed to contribute only to the tendinous slip associated with the second digit, while also inserting on the FHL tendon itself (Figure 3A). The lateral head of QP inserted only on the tendinous slip to the third digit. From a dorsal perspective, FHL was revealed to be the primary contributor to both tendinous slips (Figure 3B). These tendinous slips merged with the tendons of FDL distal to the lumbrical muscles.

## 3. Discussion

Tendinous slips arising from both QP and FHL to insert distally on FDL near the metatarsophalangeal joints have not previously been reported. This is unlike other cases in which the variant tendinous slips were strictly associated with the tendons of FDL and the lumbricals [1,6]. Additionally, a portion of QP inserted on the FHL tendon only, rather than on both FDL and FHL, as previously described in the literature [1,6].

**Figure 4:**
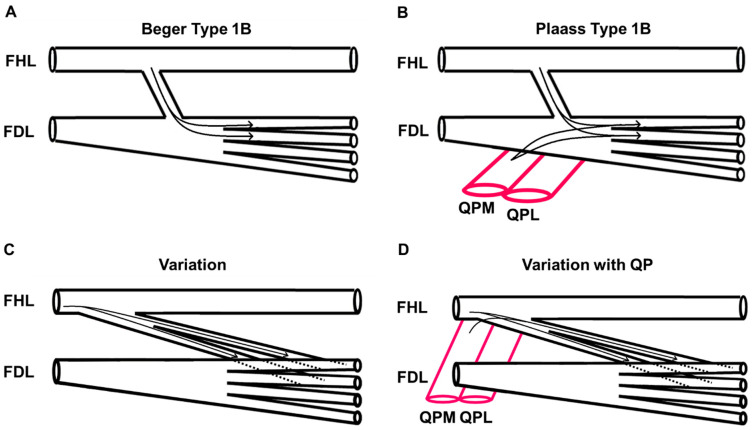
Comparison to previously described tendinous connections between FHL and FDL. Beger’s Type 1B classification (**A**) shows fibers from FHL inserting on the medial two tendons of FDL. Plaass’s Type 1B classification (**B**) shows fibers of both FHL and QP inserting on the medial two tendons of FDL. The observed variation (**C**) shows the tendinous slips of FHL inserting on the medial two tendons of FDL. Additionally, in the observed variation (**D**), the medial head of QP contributes fibers to the first tendinous slip of FDL. (Flexor digitorum longus: FDL, flexor hallucis longus: FHL, medial head of quadratus plantae: QPM, lateral head of quadratus plantae: QPL).

This case is distinct from previously reported variations of the chiasma plantare. The variations described in this report are most similar to Plaass’s Type Ib classification and Beger’s Type 1B classification in that the FHL contributes to the FDL tendons of digits two and three (Figure 4) [7,8]. However, this case is novel in that QP inserted on the FDL tendon proximal to its division into digital tendons. Furthermore, QP contributed to the tendinous slips of FHL prior to merging with the individual FDL tendons.

In the present case, it appears that QP acts solely to assist in flexion of the second and third digits. This is consistent with the theory that the muscle is primarily recruited during plantar flexion, in which FDL is contracted but further flexion of the digits may be required [13]. This is functionally relevant during the late stance phase of locomotion, in which the foot is plantarflexed and the digital extensors are active. In this posture QP would serve to resist extension of the toes, keep them in contact with the ground, and thereby enhance their stability to accept the transfer of weight. Other studies hypothesized that QP acts to either counter the oblique pull of FDL or stabilize the lumbrical muscles [1,4,6]. However, these actions would not be viable in this case given that the insertion of QP via the tendinous slips occurs distal to the division of the FDL tendon.

## 4. Conclusions

This unique case of QP both inserting on FHL and producing additional tendinous slips in conjunction with FHL is of functional significance, as this supports the proposed roles of QP as a flexor of the digits independent of FDL. While this variation would be expected to produce normal function of the toes, it could have clinical implications if the FHL tendon were to be harvested for a tendon graft, such as in Achilles rupture repair [11,14]. Harvesting this FHL tendon would disrupt then tendinous slips through which QP acts, which could result in a functional deficit in flexion of digits two and three. This case report adds to the literature regarding variations of the QP muscle, the interconnections between it and the FHL and FDL tendons, as well as the proposed actions of QP.

## Data Availability

Not applicable.
